# Molecular assessment of aortic aneurysm wall integrity using an elastin-specific MR imaging probe

**DOI:** 10.1186/1532-429X-15-S1-O4

**Published:** 2013-01-30

**Authors:** Marcus R Makowski, Andrea Wiethoff, Ullrich Ebersberger, Ulrike Blume, Alice Warley, Christian Jansen, David C Onthank, Richard R Cesati, Reza Razavi, Michael Marber, Tobias Schaeffter, Simon P Robinson, Rene M Botnar

**Affiliations:** 1 Division of Imaging Sciences, King's College London, London, UK; 2 BHF Centre of Excellence, King's College London, London, UK; 3 Philips Healthcare, Guildford, UK; 4 Cardiovascular Division, King's College London, London, UK; 5 Centre for Ultrastructural Imaging, King's College London, London, UK; 6 Lantheus Medical Imaging, North Billerica, MA, USA; 7 Department of Radiology, Charite, Berlin, Germany

## Background

The incidence of abdominal aortic aneurysms (AAAs) has increased in western societies and complications often lead to life threatening events. There is still controversy regarding the management of medium sized AAAs. Therefore, novel biomarkers, besides aneurysmal diameter, are needed to assess aortic wall integrity and risk of rupture. Elastin is the key protein for maintaining stability of the aortic and aneurysmal wall. Elastin degradation due to inflammation and metalloproteinases (MMPs) expression as well as de novo synthesis of immature elastin are considered key events in the development of AAAs.

The aim of this study was to test a novel small-molecular-weight elastin-specific MR probe for the in vivo assessment of arterial wall integrity in AAAs.

## Methods

ApoE-/- mice were infused with angiotensin II for up to 4 weeks (1000ng/kg/min). An elastin-specific MR probe (Lantheus Medical Imaging, USA) was administered 1-4 weeks following Ang-II infusion. Mice were scanned at each time point pre, post control agent (Gd-DTPA) and after administration of the elastin-specific probe. Imaging was performed using a 3T Philips Achieva MR scanner with mice positioned on top of a microscopy coil. For the assessment of contrast agent distribution, delayed enhancement (DE) MRI scans were preceded by a 2D-Look-Locker to determine the optimal inversion time for blood signal nulling. Imaging parameters of the IR-3D GE DE-MRI were: FOV=30mm, matrix=300, spatial resolution=0.1, 0.5mm slice thickness, TR/TE=28/8.2ms, TR between IR pulses=1000ms and flip angle=30°. Immediately after the DE-MRI scan, a 3D-T1 mapping sequence was performed, consisting of 2 inversion-recovery prepared modified Look-Locker trains. Aneurysmal aortic wall samples were analysed ex vivo by inductively coupled plasma mass spectroscopy and histological staining.

## Results

**Figure 1:**
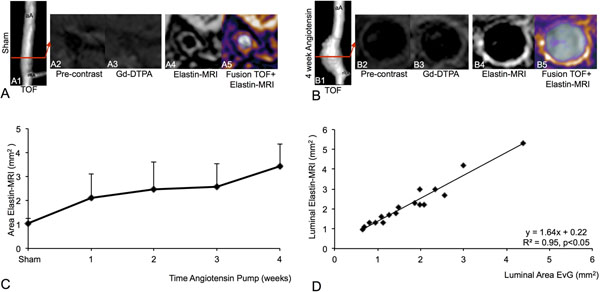
In vivo and ex vivo assessment of AAA area. A: TOF-angiogram (A1) of the suprarenal part of the abdominal aorta 4 weeks after infusion of saline in an ApoE-/- mouse (sham, control). The red line indicates the imaging plane. A non-dilated aortic lumen was found on the angiogram and elastin MRI scan (A4, A5). B: Angiogram (B1) of the suprarenal part of the abdominal aorta 4 weeks after the infusion of angiotensin II in an ApoE-/- mouse (4 week group). On the TOF, a large suprarenal aortic aneurysm could be clearly visualized. On Elastin-MRI a significantly enlarged aortic lumen and a strong enhancement of the aortic wall was visualized (B4, B5). No significant enhancement of the aortic wall could be measured after administration of nonspecific Gd-DTPA. C: A significant increase in aortic vessel wall area was observed over the 4-week time course of angiotensin II infusion. D: In vivo area measurements of lumen area on elastin MRI scans significantly correlated with ex vivo measurements on histology.

**Figure 2:**
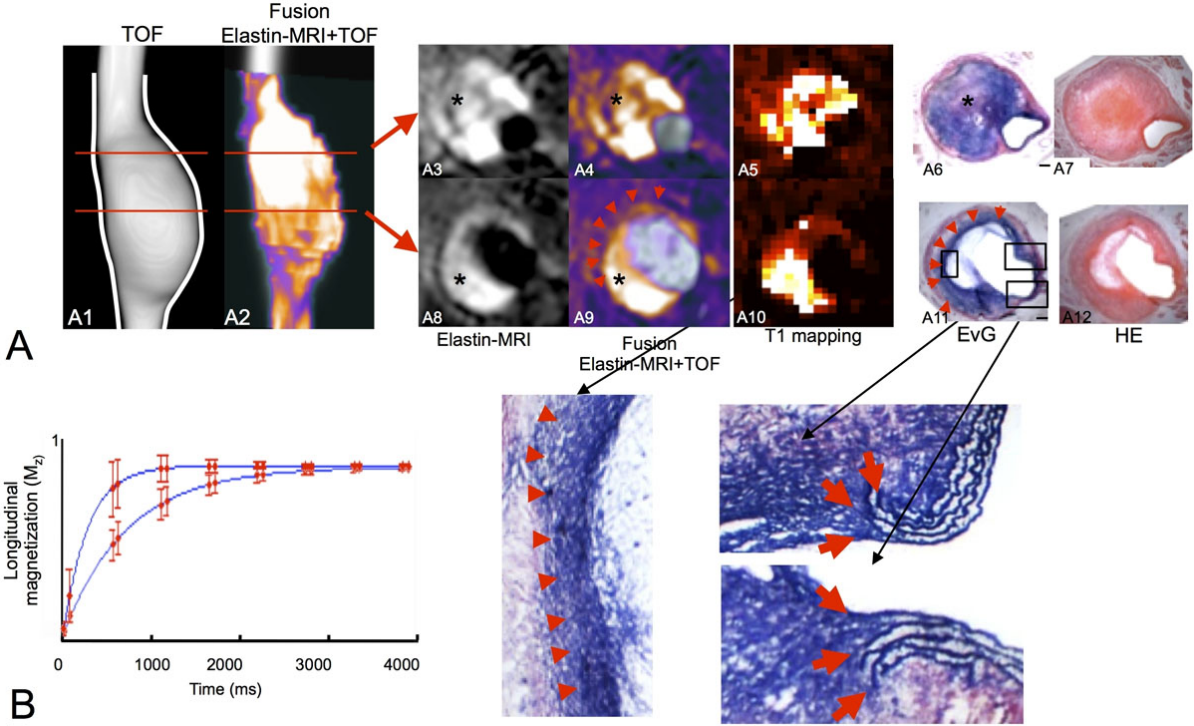
In vivo assessment of advanced thrombus remodeling. A: TOF-angiogram of the suprarenal part of the abdominal aorta (A1, A2 fusion with Elastin-MRI scan) 4 weeks after infusion of angiotensin II. On the TOF angiogram the formation of a large aortic aneurysm can be observed. At this late stage the thrombus in the aneurysmal wall is almost completely remodelled with elastin (A6, A11). The locations of rupture of the elastic laminae are still visible (A11, magnification on right side, red arrows). A "novel elastic lamina" is formed on the luminal side of the arterial wall (A11, magnification on left side, red arrow heads). A strong signal can be observed in the area of remodeling on Elastin-MRI (A3-5, A8-10, red arrows). B: Example of a T1 relaxation curve prior to and after administration of the elastin-specific MR imaging probe. A significant shortening of the T1 relaxation time could be measured post injection of the elastin-specific MR imaging probe.

The high signal provided by the elastin-specific MR probe allowed for imaging with high spatial resolution (100μm). In vivo AAA area measurements were in good agreement (R2=0.95, p<0.05, Fig. 1) with ex vivo measurement on histology (ElasticavanGiesson stain, Fig. 1). Contrast-to-noise-ratios (CNR) and R1 relaxation rates were in good agreement with ex vivo histomorphometry (Elastica stain, R2=0.77, p<0.05) and Gd concentrations determined by inductively coupled plasma mass spectroscopy (R2=0.74, p<0.05). Changes in elastin content could be readily delineated and quantified at different stages of AAAs. The most significant increase in elastin accumulation was observed in late stage aortic aneurysms (Fig. 2) and is likely related to compensatory remodeling and de novo synthesis of elastin fibers.

## Conclusions

MRI in combination with an elastin-specific MR probe offers potential for the non-invasive characterisation of the aneurysmal aortic wall during development of AAAs and may help guiding treatment decisions.

## Funding

The MRI scanner is partly supported by Philips Healthcare. A Wiethoff is an employee of Philips Healthcare. D Onthank, R Cesati and S Robinson are employees of Lantheus Medical Imaging. The study was funded by the British Heart Foundation (PG/09/061) and the contrast agent was provided by Lantheus Medical Imaging. Otherwise, there are no financial or other relations that could lead to a conflict of interest.

